# SEED-G: Simulated EEG Data Generator for Testing Connectivity Algorithms

**DOI:** 10.3390/s21113632

**Published:** 2021-05-23

**Authors:** Alessandra Anzolin, Jlenia Toppi, Manuela Petti, Febo Cincotti, Laura Astolfi

**Affiliations:** 1 Athinoula A. Martinos Center for Biomedical Imaging, Department of Radiology, Massachusetts General Hospital, Harvard Medical School, Boston, MA 02129, USA; 2 Department of Computer, Control, and Management Engineering, Sapienza University of Rome, 00185 Roma, Italy; jlenia.toppi@uniroma1.it (J.T.); manuela.petti@uniroma1.it (M.P.); cincotti@diag.uniroma1.it (F.C.); laura.astolfi@uniroma1.it (L.A.); 3 IRCCS Fondazione Santa Lucia, 00179 Rome, Italy

**Keywords:** simulated neuro-electrical data, EEG, ground-truth networks, brain connectivity, multivariate autoregressive models, partial directed coherence

## Abstract

EEG signals are widely used to estimate brain circuits associated with specific tasks and cognitive processes. The testing of connectivity estimators is still an open issue because of the lack of a ground-truth in real data. Existing solutions such as the generation of simulated data based on a manually imposed connectivity pattern or mass oscillators can model only a few real cases with limited number of signals and spectral properties that do not reflect those of real brain activity. Furthermore, the generation of time series reproducing non-ideal and non-stationary ground-truth models is still missing. In this work, we present the SEED-G toolbox for the generation of pseudo-EEG data with imposed connectivity patterns, overcoming the existing limitations and enabling control of several parameters for data simulation according to the user’s needs. We first described the toolbox including guidelines for its correct use and then we tested its performances showing how, in a wide range of conditions, datasets composed by up to 60 time series were successfully generated in less than 5 s and with spectral features similar to real data. Then, SEED-G is employed for studying the effect of inter-trial variability Partial Directed Coherence (PDC) estimates, confirming its robustness.

## 1. Introduction

In the last decade, the interest in brain networks has grown in the world of neuroscience [1,2,3,4,5]. Connectivity estimators are powerful tools to determine which brain areas are mainly involved in the execution of motor and cognitive tasks, and how they communicate to generate the complex brain networks underlying specific cerebral functions. Several connectivity estimators with different features have been developed to assess the existence, the intensity and the direction of the statistical connections linking two or more time series. Some of them are highly versatile as they can be applied to signals acquired through different techniques such as functional magnetic resonance imaging (fMRI), electroencephalography (EEG) and magnetoencephalography (MEG) [6,7]. The ensemble of connectivity estimators is a manifold scenario where the selection of the appropriate algorithm on the basis of the type and quality of the data and of the research objectives is not trivial. Several studies previously compared the performances of existing algorithms under different experimental conditions with the aim to optimize this choice [8,9,10,11,12,13]. Additionally, new algorithms and adaptations of existing ones should always be validated and compared to previous methods in order to define guidelines for their best use. In both cases, the quality assessment of the connectivity estimates requires time series with a known underlying connectivity pattern (ground-truth network), which can be used as a test bench.

Only a few physiological patterns, uncovered by widely replicated findings and anatomical evidence can be used for the validation of connectivity methods on real data. Mill et al. employed an associative memory task to elicit and isolate connectivity from visual to auditory areas and in the opposite direction to validate functional connectivity in MEG and fMRI. In this example, the ground-truth was hypothesized on the basis of known anatomical interconnectivity in animals [14], similarly to what was done in [15]. Although this approach allowed for testing of different connectivity estimators in a real context, it comes with several limitations such as the lack of information about the intensity of the information flow, which does not allow for assessing the accuracy in the magnitude estimate, and restrictions in terms of model structure and dimension.

Another reliable solution for the quality assessment of connectivity estimates and validation is to simulate datasets with a known connectivity pattern (ground-truth). Benchmark datasets arise from two main steps, the ground-truth connectivity pattern design, and the signal generation. This process still represents a weak point in the testing of connectivity methods as only a limited set of realistic conditions has been modeled. One critical aspect of the data generation lies in the definition of several parameters of the ground-truth network, such as the number of nodes (simulated electrodes/brain regions), network density (number of connections) and network global properties that can be chosen. Furthermore, the construction of the signal should preserve the spectral properties of the real data.

The generation of stationary and time-varying ground-truth networks has been previously proposed and successfully applied to compare different methods, but always with specific limitations. One proposed solution used time series generated from imposed linear toy models whose equations are designed so that the first signal behaves as an oscillator driving the other structures [16,17]. Another attempt constructed signals from a multivariate autoregressive (MVAR) model used as generator filter where white noise is given as input and the model coefficients are manually imposed by the experimenter [18,19,20]. Neither of these approaches allow for the generation of realistic signals because their spectral properties do not replicate the ones observed on real neuroelectrical time series. In order to generate more realistic time series, a cascade approach has been proposed where a real EEG signal is selected as the main source and all of the other signals are iteratively obtained from it according to a ground-truth connectivity pattern [2,5,12,20,21]. The main limitation of the cascade method is that it cannot be employed to generate datasets with a high number of signals since it leads to an incontrollable increase of the signals’ amplitude. Wang et al. compared several connectivity methods applied to EEG time series: a convolution-based neural mass model along with another three models to take into account the fact that various algorithms for functional connectivity are differently sensitive to non-linearities in the data [11,22]. The generative models in question are linear systems, non-linear systems with linear coupling, and non-linear systems with non-linear coupling, while the considered connectivity models are all possible topologies for only five nodes and five edges. More information about the optimal use of different estimators in different conditions could be gained including bigger and more flexible models.

Some free packages for connectivity estimation that have been released include functions for the generation of simulated data for testing purposes. The Multivariate Granger Causality (MVGC) toolbox developed by Barnett and Seth simulated multi-trial data from normally distributed residuals for given VAR parameters [23,24]. More recently, the toolbox by Haufe and Ewald proposed the generation of pseudo-EEG (and MEG) data with an imposed directed interaction in which the time courses of two distinct sources at cortical level are modeled using bivariate linear autoregressive models [25]. Although those tools played an important role in the investigation of specific aspects of brain connectivity analysis pipelines such as source reconstruction algorithms and connectivity matrices validation [8], they still present limitations. The MVGC toolbox permits the generation of datasets with a higher number of signals compared to those previously listed; however, the generated time series do not replicate the typical EEG spectral properties. On the other hand, the toolbox proposed by Haufe and Ewald can simulate pseudo-EEG data including EEG-like spectral properties and spurious effects created by volume conduction, but only between one target and one driver. The main goal of their study was to provide a benchmark limited to a minimal realistic case of brain interaction that should be extended to model more complex cases, which is one of the aims of the present manuscript.

Another interesting aspect to model in the connectivity ground-truth is the non-stationarity of the functional links between brain regions. In order to test the increasingly popular dynamic connectivity algorithms, ground-truth connectivity models must be capable of representing non-stationarity. Previous studies either employed a small model (less than five nodes) with some time-dependent connections modeled as a step function [13,20] or, in one specific case [21], a more complex eight-node time-varying model was built to simulate the reorienting system of the human brain theorized in [26].

In general, what is currently lacking in the available toolboxes/approaches to simulate pseudo-EEG signals is the ability to generate datasets (i) with a given dimension, able to match high-density EEG configurations, (ii) with the spectral properties of the real signals, (iii) organized as a long single-trial or as a multi-trial dataset, and (iv) including non-idealities, like artifacts (e.g., eye-blinks) and realistic conditions in which the pattern is non-stationary or inconsistent across the trials.

The main aims of our study were (i) to release a new, free, and easy-to-use toolbox called SEED-G (Simulated EEG Data Generator), (ii) to show its capability to overcome these limitations and facilitate the quality assessment of connectivity methods in several scenarios, and (iii) to test its performances, providing guidelines for potential users and future studies. In fact, SEED-G allows for generating realistic simulated datasets with the additional functionality of tuning the properties of the time series as well as the features of the connectivity model. The enhanced control over several parameters allows SEED-G to provide simulated time series molded to the user’s needs and flexible enough to model more than one standard experimental condition. The features that can be adjusted include the length of the time series (number of samples), the number of trials composing the dataset, the signal to noise ratio (SNR), inclusion or not of non-brain artifacts (ocular blink), as will be detailed in the following paragraphs. Moreover, this toolbox permits the configuration of several parameters of the imposed connectivity pattern, including the brain network dimension (number of nodes), the network density, the temporal dynamic (stationary or time-varying) and the presence or not of non-idealities (inter-trials variability) in order to obtain a ground-truth in many different and not yet investigated experimental conditions.

The manuscript is structured as follows: we begin with a detailed description of the SEED-G functionality, followed by an evaluation of the toolbox performances in terms of computational time and quality of the generated time series. The second part of the paper is devoted to portraying an example of possible application of SEED-G, namely the testing of connectivity estimators under non-ideal conditions.

To this purpose, we used SEED-G toolbox to generate simulated datasets fitting connectivity patterns that vary across trials, with the aim to assess how inter-trial variability affects accuracy and consistency of the well-known MVAR-based estimator, Partial Directed Coherence (PDC) [16]. The non-idealities across trials were modeled in two different ways, where the connectivity links were altered in value or number, respectively. Preliminary data were published in [10], but the results were limited to 10-node models. We propose here a more extensive evaluation of this phenomenon exploiting several more experimental conditions simulated using the SEED-G toolbox.

SEED-G toolbox was developed in MATLAB environment and is publicly available at https://github.com/aanzolin/SEED-G-toolbox (accessed on 12 April 2021).

## 2. Toolbox Description

In the proposed toolbox, the generation of pseudo-EEG data that reproduce well-known ground-truth networks is performed using multivariate autoregressive (MVAR) models as generator filters to provide a benchmark for functional connectivity estimates. SEED-G toolbox functionalities include:

1. The generation of a predefined ground-truth connectivity model with:
  - a selected size (number of signals to be generated);
  - a selected density;
  - parameters randomly assigned within a given range;
  - stationary or time-resolved connectivity values.
2. The generation of pseudo-EEG time series with:
  - spectral similarity to reference EEG scalp- or source-level data;
  - given length in terms of number of samples;
  - number of trials;
  - predefined SNR;
  - inter-trial variability;
  - presence of ocular artifacts.

**Figure 1:**
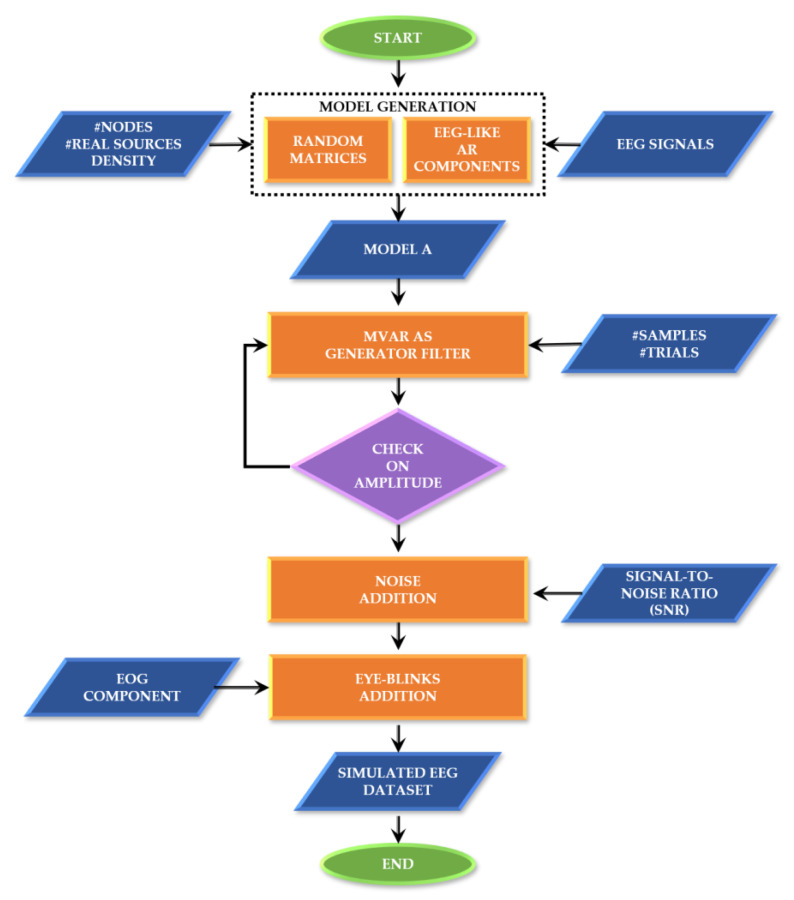
SEED-G generator framework. Block diagram describing the main steps at the basis of the data generator.

These functionalities and their testing will be described in more detail in the next paragraphs, while Appendix A contains information about the corresponding MATLAB functions, dependencies and demos. As an overview, Figure 1 synthesizes the simulated EEG data generation process, including the definition of the ground-truth and the generation of the time series.

### 2.1. Simulated Data Generation

Ground-truth model generation. If not provided by the user, the toolbox first generates a ground-truth connectivity model which is then used as matrix of coefficients in the MVAR model. Alternatively, the user can provide the model as input. Ground-truth connectivity model consists of a weighted, directed 3D matrix of dimensions equal to NxNxp where N is equal to the number of nodes and p is equal to the MVAR model order. The matrix is automatically created by the software by imposing the non-null connections (whose number can be chosen by the user) equal to a value randomly selected within a specific range (chosen by the user). This value is imposed only at one lag among the possible p lags, randomly selected. The null connections are instead characterized by null coefficients at each lag.

In order to obtain simulated signals that reproduce the spectral properties of brain data, the user could include real sources in the model (scalp, cortex, or reconstructed data) as AR components on the main diagonal of the ground-truth model. The data simulated for this study used real sources extracted from real signals acquired during resting state in a healthy subject with sampling rate equal to 200 Hz. In this first phase, the parameters to be set are: the number of signals to be generated, the amount of non-null connections imposed in the ground-truth network, and the number and type (scalp/cortex) of real sources included in the model. The final number of EEG sources included in the model depends on the structure of the generated ground-truth network. When nodes in the network are not directly nor indirectly linked to one of the EEG sources (isolated nodes), the corresponding signals are replaced with real EEG sources. Thus, the number of sources included in the model is equal to the number of sources selected by the user plus the number of isolated nodes.

Time series generation. After the ground-truth network generation, a MVAR-based generator filter creates the dataset of time series. The MVAR model uses uncorrelated, zero mean innovation noise simulated through a normally distributed pseudorandom generator. The covariance of the innovation process is set equal to the identity matrix. To obtain signals as realistic as possible, the user can decide to sum to the time series output of the MVAR model, (i) an additive noise according to a predefined SNR value and (ii) a vertical ocular component simulating the blink effect (see paragraph below). The user can set the following parameters: number of data samples for each realization, number of realizations to be generated and SNR values. Noise-free signals could be generated by setting SNR to infinity value. For each generated time series, a threshold criterion is applied to check amplitude of generated time series. If the amplitude of a simulated signal exceeds in absolute values the threshold (automatically set to 80μV), the trial is rejected, and its generation repeated.

### 2.2. Realistic Features Modeling

Inter-trial variability. In the case of multi-trial dataset, the inter-trial variability typical of real data can be modeled in the ground-truth. The SEED-G toolbox models two kinds of non-idealities across trials: (i) variability of the intensity of existing connections hypothesizing that connections values are not consistent across repetitions and (ii) variability in network density in order to account for the presence of spurious connections in some trials. The amplitude of these non-idealities can be regulated by choosing the percentage of altered trials, the number of connections to be modified in weight, the entity (percentage with respect to original weight) and the direction (increase or decrease with respect to the original weight) of the variation, and the number of spurious links added to the original model in specific trials.

**Figure 2:**
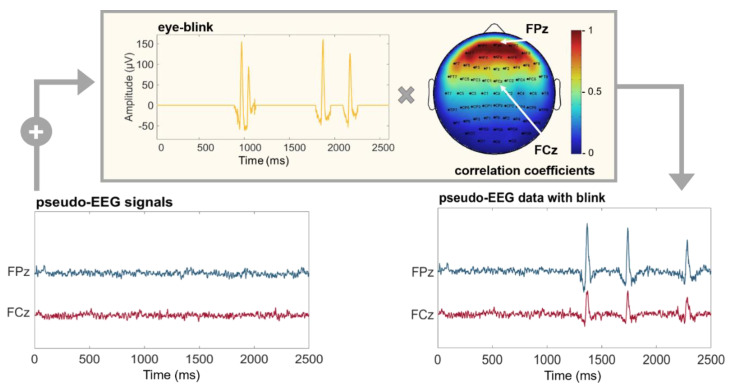
Pseudo-EEG data with ocular artifacts. Time series simulated using SEED-G toolbox (pseudo-EEG signals) and real ocular blinks (eye-blink) were summed up to obtain a realistic dataset (pseudo-EEG data with blink). The eye-blink component was weighted by a correlation coefficient whose value depends on the position of the simulated sensor (Pearson’s correlation between real EEG dataset acquired with a standard 60-channel montage, and an EOG channel). Frontal electrodes are maximally affected by the presence of the blinks; indeed, the correlation coefficient is equal to 1 for the electrode FPz (red portion of the mask). The same coefficient is equal to 0.5 for the sensor FCz, reflecting the fact that more central sensors show attenuated ocular components.

Ocular component. Real EEG data are often contaminated by ocular artifacts, especially when recorded from frontal positions. These kinds of artifacts could affect the performances of connectivity estimators under examination and should be included in the time series used as a benchmark. SEED-G can simulate the blink component by adding a real ocular component to the generated time series with a specific amplitude depending on the position of the electrodes. More specifically, the weight was extracted from a mask obtained correlating an electro-ocular signal (EOG) with the EEG acquired from each sensor of a 60-channel standard net (Figure 2).

Time-varying networks. SEED-G toolbox permits the generation of stationary or time-varying connectivity ground-truths. The generation process is the same, but in the dynamic case, a different connectivity model is imposed for each time point. It is possible to choose between two kinds of temporal transitions according to either a step or a ramp profile. In the step transition, the same ground-truth model is retained for a predefined number of samples, after which some of the connections instantly change their weight while the others keep the original value. This new pattern holds until the end of the observation window. In the ramp profile, the same transition happens gradually instead of instantaneously. Given a selected transition window, the connections’ weights vary linearly within it. The variables set by the user are the percentage of connections varying across time, the type of transition, the instant in which a transition starts, the instant in which a transition ends (only if the ramp profile is selected), and the magnitude of the connectivity weight variations expressed as percentage with respect to the pre-transition value.

Generation of signals at the source level. Depending on the type of real sources used for the signal generation, SEED-G can simulate both scalp and cortical data. In the latter case, by default, the toolbox uses source data, reconstructed using the software sLORETA [27] from a real EEG signal recorded from a healthy subject (64 channels and sampling rate equal to 200Hz). Real ECoG data can also be used as sources by the user, if available. Additionally, generating data with an imposed pattern at source level and projecting them at scalp level allows to take into account the effect of the brain volume conduction on scalp signals. The forward model included in the toolbox is the New York Head available on the ICBM-NY platform, which includes 231 sensors on the scalp and 75,000 nodes on the cortical surface [28].

## 3. Evaluation of SEED-G Toolbox Performances

In this section, we proposed a simulation study with the twofold aim of demonstrating capability and versatility of the toolbox SEED-G and providing guidelines for its optimal use in different conditions. For this purpose, we simulated datasets with different features, described in more detail in the following sections, and we reported the distribution of meaningful performance parameters quantifying the time necessary to successfully complete the generation of a dataset, the number of failed attempts (especially for models with high number of nodes), and the spectral quality of the generated time series.

### 3.1. Methods

We generated simulated datasets by systematically imposing different values of factors like model dimension, network density and number of real sources, and defined for each of them specific performance parameters. Each isolated node was associated to a real source (resting EEG signal from a healthy subject with sampling rate equal to 200 Hz) and each connection was considered invariant across samples and trials. To increase the reliability of the results, the entire procedure of data generation and performance evaluation was repeated 300 times.

#### 3.1.1. Pseudo-EEG Time Series Generation

In order to reproduce realistic EEG datasets, we imposed parameters spanning a range typical of real experiments:

- “Model size” is the number of time series composing the dataset. Levels: (5, 10, 19, 32, 60) nodes, simulating a range between few electrodes and the most commonly used extended 10–20 scalp EEG montage.
- “Network density” is the percentage of non-null coefficients. Levels: (5%, 10%, 20%, 30%) of the possible connections.
- “Real sources” are the percentage of real sources included as sources in the model with respect to all generated signals. Levels: (20%, 30%, 50%) of the number of the generated time series.

#### 3.1.2. Performance Parameters

Extra Required Iterations (ERIt). Given that every time a simulated signal exceeds 80 µV of amplitude it gets discarded, and an extra iteration is required, ERIt is defined as the number of rejected simulated datasets, normalized by the maximum number of iterations allowed. ERIt is equal to 0% if the dataset is generated without extra iterations and it is equal to 100% when the dataset was generated using the maximum number of iterations (which is 1000 in this simulation study) and quantifies how easily a dataset was generated for a specific combination of parameters.

Computational Time: time in seconds required for the generation of a complete dataset using an Intel Core i5 3.2GHz CPU, 16GB RAM.

EEG-like Signals (EEGl_S%): percentage of simulated signals showing EEG-like spectral properties. The spectral similarity between pseudo- and real EEG signals was quantified by performing a Pearson’s correlation between the Power Spectral Density (PSD) of each simulated data and the PSD of one of the real sources. PSD was estimated using the Welch’s method with Hann window of 1 s length. The generated time series were considered as an EEG-like signal if such correlation is higher than 0.6. The percentage of signals of realistic data was computed as:

(1) $$EEGl\_S_{\%}=\frac{n_{X}-n_{AR}}{N-n_{AR}}*100$$

where $n_{X}$ is the number of EEG-like signals, $n_{AR}$ is the number of the MVAR real sources (with an AR component different from zero), and *N* is total number of generated signals.

*Descriptive Analysis:* the distributions of the performance parameters computed for each of the 300 iterations were reported in boxplots for each simulated condition (combination of “Model size”, “Network density” and “Real sources”).

### 3.2. Results

In this section, we report the performance achieved by the SEED-G data generator across 300 iterations in terms of extra required iterations (if needed for the generation process), computational time, and spread of the spectral properties from the real source.

**Figure 3:**
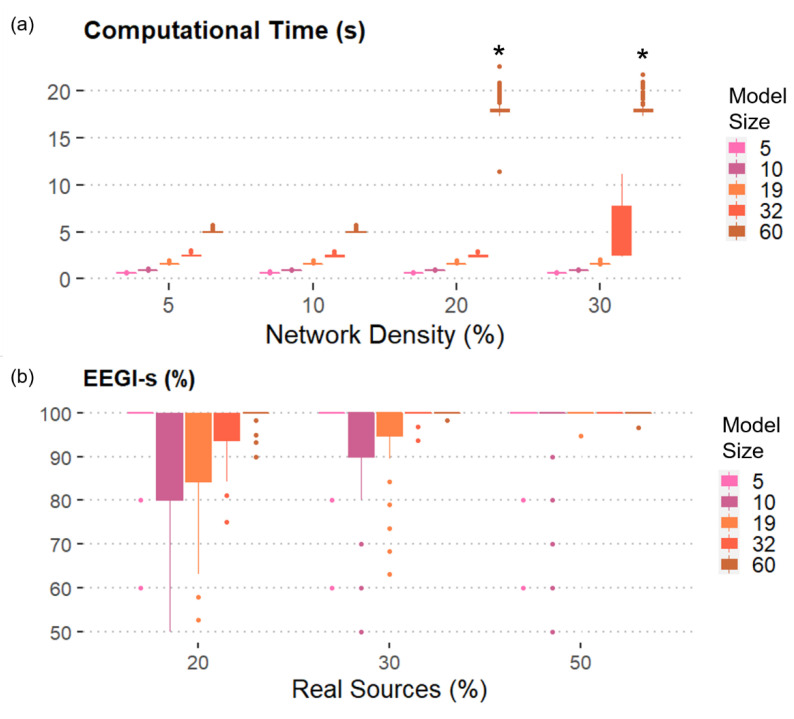
SEED-G performances in terms of computational time and percentage of realistic data generated: (**a**) Mean and standard deviation of the computational time computed over 300 iterations were shown as functions of the number of time series (Model size) and the number of existing connections (Network density). The number of real sources included is equal to 30%. Except for some 60-node models, all the simulated datasets composed by 100 trials were generated in less than 10 s. (*) encodes the fact that no dataset with that specific combination of features could be generated with 1000 attempts; (**b**) Mean and standard deviation of the percentage of EEG-like signals (*EEGl*-s) computed over 300 iterations were reported as functions of Model size and of the number of real sources included in the connectivity model (Real sources). The density of the network is equal to 10%. On average, more than 80% of the generated data reported EEG-like spectral properties.

Figure 3a reports the results obtained when the number of real sources included in the model was set equal to 30% of the total number of generated signals. As expected, we found that the computational time and the ERIt increased with the model size and the network density. However, the toolbox could generate simulated EEG dataset with up to 60 nodes without extra iterations and in less than 5 s when the density of the network is 5% or 10%. In case of 30%-dense connectivity patterns, it was possible to generate datasets composed by 32 linked time series with 25% of extra iterations required on average (see Figure S1) in less than 10 s. The most complicated case to simulate was the one with 60 pseudo-EEG signals connected by a statistical network whose density was greater or equal than 20%. Such dataset was not generated even after using all the 1000 attempts. Similar results were obtained by changing the percentage of real sources in the model to 20% and 50% of the total number of time series included in the generated dataset, demonstrating that the number of real sources did not influence the number of extra iterations and the computational time required for the generation process. The results obtained for 20% and 50% of Real sources were reported in the Supplementary Materials, along with the graphs relative to the extra required iterations.

We also reported the percentage of EEG-like signals when the network density is equal to 10% in order to show the results for all the levels of the factor Model size (Figure 3b). More than 80% of the simulated signals showed real EEG spectral properties, regardless of the other factors, including the number of real AR components included in the model. As expected, an even higher percentage of EEG-like signals was generated when either more connections or more real sources were considered.

## 4. SEED-G Toolbox Application: Evaluation of the Inter-Trial Variability Effect on PDC Estimates

**Figure 4:**
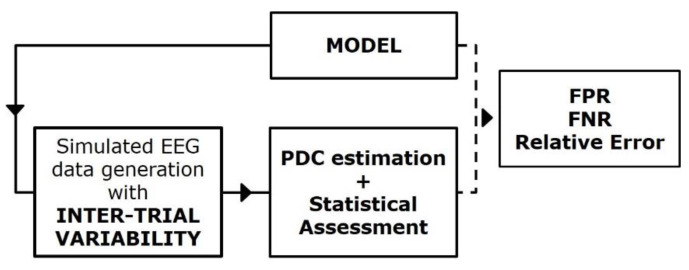
Simulation study for the PDC robustness assessment. Block diagram describing the simulation framework built for quantifying the effect of the inter-trial variability on connectivity estimates’ performances.

Figure 4 reports a block diagram describing the principal steps of the simulation framework built for studying the effect of inter-trial variability on PDC consistency and accuracy [16,17]. In this context, SEED-G was employed for simulating pseudo-EEG datasets fitting ground-truth networks non-consistent over trials. The inter-trial variability was imposed separately in two different ways:

- by increasing/decreasing the value of some existing connections in the ground-truth network (Study I);
- by modifying the ground-truth network density by adding some spurious connections to the existing ones (Study II).

The parameters imposed for the data generation and the evaluated performances are described in detail for each study in the following paragraphs.

### 4.1. Methods

#### 4.1.1. Study I: The Effect of Unstable Connectivity Values

Several pseudo-EEG datasets were generated for the analysis using the SEED-G toolbox after setting the following parameters:

- Model size: 5, 10, 20 nodes;
- Network density: 20% of the possible connections;
- Connections’ intensity: randomly selected in the range [−0.5:0.5];
- Percentage of modified trials: 1, 10, 30, 50% of the total number of the generated trials;
- Percentage of modified links across-trials: 10, 20, 50% of existing connections;
- Amplitude of the variation: 20, 50, 70% of the original value of the connection;
- Type of variation: positive (increase), negative (decrease).

Connectivity values were estimated on the simulated data using the PDC and assessed against null-case by means of the asymptotic statistic approach with a significance level α equal to 0.05 corrected by False Discovery Rate (FDR) to reduce family-wise error rate due to multiple comparisons [29,30,31].

The quality of the results was evaluated in terms of *Relative Error*, defined as the sum of all the relative prediction errors obtained for each connection in the pattern (where $N_{c}$ is the total number of connections). The magnitude of relative prediction error for the specific connection i is obtained as the Frobenius norm of the difference between the estimate averaged across frequencies $(PDC_{i}$) and the imposed connection value $(Model_{i}$) normalized for the imposed value:

(2) $$Relative\;Error=\sum_{i=1}^{Nc}\left|\frac{PDC_{i}-\left|Model_{i}\right|}{\left|Model_{i}\right|}\right|$$

Finally, in order to differentiate the effect of the alterations in the connections’ value on the estimation in different experimental conditions, we performed a four-way ANOVA with the relative error as dependent variable, Tukey’s post hoc for testing differences between sublevels of ANOVA’s factors and Bonferroni–Holm correction for multiple ANOVAs. The main within factors were the percentage of modified connections (MOD_CON), the entity of the variation (VAR), the direction of the variation (VAR_DIR), and the percentage of modified trials (TRIALS). In order to increase the robustness of the statistical analysis, the entire procedure was iterated 100 times, with a different ground-truth at each repetition of the simulation process. The statistical analysis was performed using Statistica 8.0 software for Windows (StatSoft, Inc.).

#### 4.1.2. Study II: The Effect of Spurious Connections

Similar to Study I, pseudo-EEG datasets were generated for the analysis using the toolbox under different settings:

- Model size: 5, 10, 20 nodes;
- Network density: 20% of the possible connections;
- Connections’ intensity: randomly selected in the range [−0.5:0.5];
- Percentage of modified trials: 1, 10, 30, 50% of the total number of trials generated;
- Percentage of added spurious links: 10, 20, 30% of all existing connections.

Consistent with the previous analysis, PDC was employed to estimate the connectivity values between each pair of generated signals and the asymptotic statistic approach for their validation (α equal to 0.05, FDR corrected) [29,30,31].

The quality of the functional connectivity performances was then assessed by computing the False Positive Rate (FPR) and the False Negative Rate (FNR). FPR is the percentage of false positives obtained by comparing the estimated networks with the imposed ground-truth in terms of null and non-null connections. Estimated patterns were averaged across the entire frequency range 1–45 Hz, binarized according to the application of null-case statistical threshold and then compared with ground-truth. False positives were defined as the connections that resulted as significant from the estimation/validation process while their value in the model was set to zero. The amount of these links was then normalized on the number of all the possible false positives ($N_{null}$) in order to obtain the FPR as:

(3) $$FPR=\frac{1}{N_{null}}\underset{n\in N_{null}}{\sum }K^{+}\left[n\right]$$

where $N_{null}$ is the total number of null connections and $K^{+}\left[n\right]$ is 1 if PDC evaluated on null arcs’ value is above threshold of significance and 0 otherwise.

The FNR instead, is the percentage of false negatives obtained by comparing the estimated binary networks with the imposed ground-truth. FNR takes into account the connections that resulted as non-significant in the PDC estimation while their value was different from zero in the ground-truth. The amount of these links was then normalized on the number of all the possible false negatives, thus on all the existing links $(N_{non-null}$).

(4) $$FNR=\frac{1}{N_{non-null}}\underset{n\in N_{non-null}}{\sum }K^{-}\left[n\right]$$

where $N_{non-null}$ is the total number of existing connections and $K^{-}\left[n\right]$ is 1 when the PDC value of non-null arcs is under statistical threshold and 0 otherwise. We also compared the effect of spurious connections on PDC estimates in different experimental conditions performing a three-way ANOVA (Tukey’s post hoc for pair-wise comparisons and Bonferroni–Holm for multiple ANOVA correction). The main within factors were the percentage of added connections (SPURIOUS) and the percentage of modified trials (TRIALS). The model size (MOD_SIZE) was the between factor. FPR and FNR were the dependent variables. In order to increase the robustness of the statistical analysis, the entire procedure was iterated 100 times, with a different ground-truth at each repetition of the simulation process. The statistical analysis was performed using Statistica 8.0 software for Windows (StatSoft, Inc.).

### 4.2. Results

#### 4.2.1. Study I: The Effect of Unstable Connectivity Values

**Figure 5:**
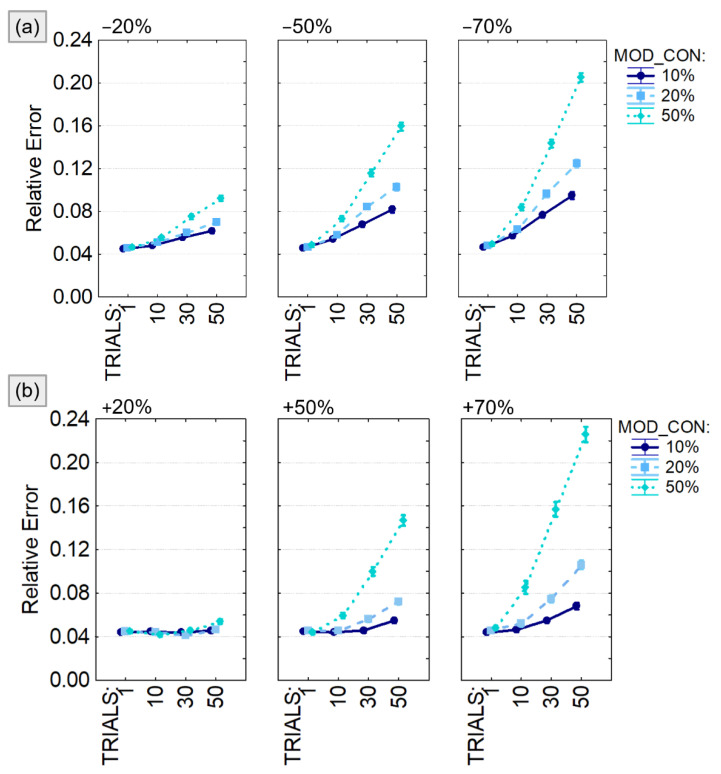
PDC percentage estimate error with respect to the imposed connectivity model. Results of the four-way ANOVA performed using Relative Error as dependent variable. The investigated factors are: the number of connections with variable weight (MOD_CON), the amplitude of the weight variation quantified as percentage of the initial value (VAR), the percentage of modified trials (TRIALS), and the type of variation (number of connections on which to apply a value variation (VAR_DIR) which can be either a reduction (panel **a**) or an increase (panel **b**). The bars represent the 95% confidence interval.

The output of the four-way ANOVA performed using the relative error as dependent variable were reported in Table 1 and showed a significant effect of all the considered factors (VAR_DIR, VAR, MOD_CON, TRIALS) and their interactions. The same analysis was repeated for different sizes of the connectivity model (5, 10 and 20 generated time series) leading to similar conclusion. For this reason, we decided to present the trend of the relative error computed only for 10-node models (Figure 5) and to provide codes and data to replicate them for the other experimental conditions. We found that the estimation error associated with the PDC computation was significantly higher when the number of modified trials (TRIALS) increases as well as the amount of modified connections (MOD_CON) in both positive (Figure 5a) and negative (Figure 5b) amplitude variations. The estimation error was lower than 10% unless the simulated connectivity variability was imposed in half of the generated trials or increased/decreased of 70% of the real value. It is notable that, even in the worst case, the relative error was lower than 25%.

#### 4.2.2. Study II: The Effect of Spurious Connections

**Figure 6:**
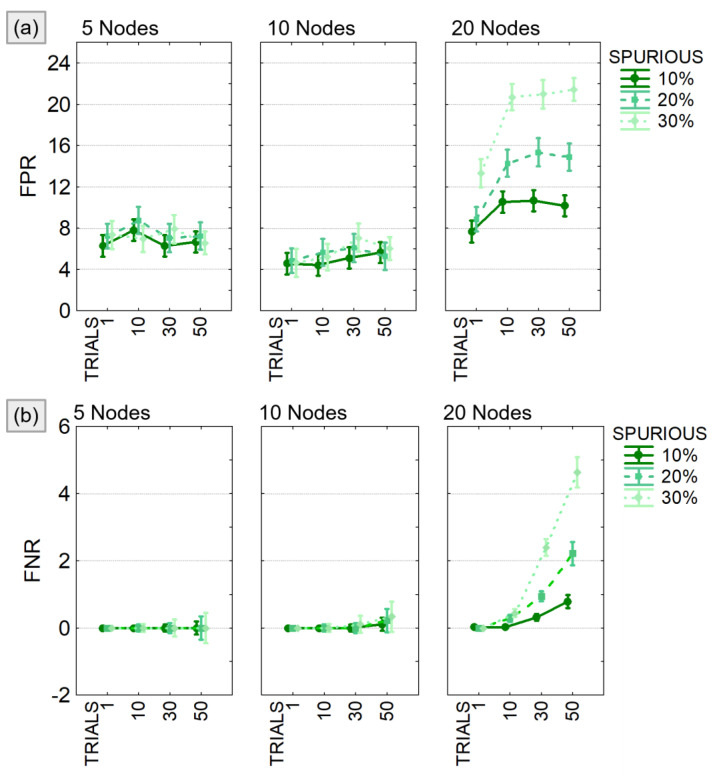
False positive and false negative in PDC estimates. Results of the three-way ANOVA performed to compare FPR (panel **a**) and FNR (panel **b**) in different experimental conditions defined by the number of existing spurious connections (SPURIOUS), the percentage of modified trials (TRIALS), and the dimension of the model (MOD_SIZE). The bars represent the 95% confidence interval.

The results of the three-way ANOVA performed to compare FPR and FNR in different experimental conditions showed a significant effect of all the considered factors (SPURIOUS, TRIALS, MOD_SIZE) and their interaction (see Table 2). As expected, both FPR and FNR increased with increasing model size and number of modified trials. When considering, at most, 10 nodes, the FPR (Figure 6a) was lower than 10% and the FNR (Figure 6b) lower than 1% regardless of the other two factors. In case of bigger models (20 nodes), both FPR and FNR showed a significant increase, and the effect of the factors SPURIOUS and TRIALS was certainly more distinct.

## 5. Discussion

In the context of the increasing importance of connectivity studies for both basic science and clinical applications, we developed an open-source toolbox called SEED-G for the validation and better understanding of new and existing estimators. Our aim was to show its capability to overcome the limitations of existing tools and facilitate the quality assessment of connectivity methods in several scenarios not addressed by previous methods.

The toolbox is indeed capable of generating “personalized” simulated pseudo-EEG datasets with imposed connectivity patterns as a benchmark for testing connectivity methods under different conditions. The strength of SEED-G resides in the flexibility of choice in both the connectivity patterns and the properties of the generated signals, to be selected according to the estimator and the scenarios to be tested. In order to perform a reliable testing procedure, whose conclusions can be effectively extended to real applications, we included within the SEED-G toolbox the possibility to simulate signals with EEG spectral properties, as well as some non-idealities, such as non-consistent connectivity patterns across the trials (simulating the repetitions of an experiment) and the presence of measurement noise or ocular artifacts. Additionally, the SNR can be modulated to identify which estimators are more robust to noise than others.

To the best of our knowledge, no other existing tools or dataset provide the capability to generate connectivity models and time series with such properties. In fact, ground-truth models of specific tasks, based on anatomical evidence in animals [14,15], are intrinsically non-flexible, while simulators of benchmark datasets previously available were able to generate either small models, with a few nodes [2,5,12,20,21,25], or data with spectral properties that do not replicate the ones of real neuroelectrical time series [16,20,21,22,23,24], and did not include non-idealities like artifacts and inter-trial variability.

Brain connectivity networks are also known to change over time under specific experimental conditions, and time-varying connectivity estimators are needed to follow those meaningful fluctuations [32]. The SEED-G toolbox allows for the generation of pseudo-EEG data fitting time-varying connectivity patterns and, consequently, for testing if dynamic estimators can catch these variations with sufficient accuracy. We believe that designing ad hoc simulation studies, as enabled by our toolbox, will help to identify weak points of several time-varying methods, and will have a positive impact on their optimization.

Still an open issue in the context of brain connectivity analyses is the effect of specific steps of the EEG pre-processing on the estimates [33,34]. Several algorithms employed for non-brain signal detection and isolation might introduce artifacts in the connectivity networks estimated between brain regions (or scalp electrodes). We suggest that SEED-G toolbox might contribute to identifying the key steps to avoid while quantifying the extension of those artifacts in increasingly non-ideal conditions. A meaningful example in this direction is the study of algorithms for the ocular component removal, like the Independent Component Analysis (ICA) and their effects, if any, on the connectivity network estimates. In this paper, we showed how the proposed toolbox generates realistic pseudo-EEG data with blinks whose magnitude depends on the spatial location of the simulated electrode over the scalp, and their frequency of occurrence can be chosen by the user to simulate different kinds of experimental subjects. More artifacts typical of the EEG acquisitions (and a combination of them) will be modeled in future releases of the toolbox, opening the way to investigate the goodness of different artifact rejection approaches and their impact on the subsequent connectivity estimates.

Even the investigation of the volume conduction effect on MVAR-based estimators, widely debated in the last years [35,36,37], requires simulated dataset fitting known connectivity models. We suggest that the generation of large (up to 60 nodes) and more complex networks will help understanding advantages and disadvantages of different head models and to test linear-inverse algorithms in combination with connectivity measures as already been done with simpler models [8,25]. In this regard, SEED-G can be used either to generate pseudo-EEG signals which will be projected on cortical and subcortical areas or, more interestingly, to generate the data and the underlying network at source level and propagate them to the scalp in order to model the volume conduction in the sensors’ space. As a side note, we suggest either using real electrocorticographic (ECoG) signals to extract the AR components of the model or real EEG data after source reconstruction when simulating signals at source level.

The SEED-G toolbox certainly presents some limitations, given that particularly large and dense networks (32/60 nodes with 30% of the possible connections) still cannot be generated as shown from the analysis of the toolbox performances. This limitation is likely a consequence of the fact that the diagonal elements of the MVAR matrices are assigned to individual AR values while the off-diagonal elements are randomly set, leading to unstable MVAR models. One of the future additions will be a test for MVAR stability which might also help in developing further functionalities with the aim to generate more computationally intensive networks.

As for the choice of a generation approach based on MVAR models, we selected it for its flexibility and its capability to reproduce the spectral properties of bioelectrical signals. With respect to other approaches, like those based on neural oscillators, MVAR models allowed for increased number of generated time series to match real data dimension, without considerable stability problems, and enabled the control of all the factors we wanted to study and manipulate in the generation process. On the other hand, its main drawback is the hypothesis of linear relationships between time series. Future releases of the toolbox may include the possibility to impose a non-linear relationship between simulated brain signals.

Other improvements could include: (i) the simulation of further non-brain artifacts other than blinks (e.g., saccade and muscular components), (ii) the refinement of the signal generation at source level including in the process features that depend on their position in the cortex, (iii) expanding the toolbox for simulating other interesting phenomena typical of the EEG data, such as even related potentials (ERP), opening the way to the validation and optimization of other algorithms.

As an application, we employed the proposed toolbox to investigate the effect of the inter-trial variability on the accuracy of the PDC estimator under realistic experimental conditions never simulated before. High variability across different trials is common in EEG, due to methodological issues (degradation of the signal quality over time) as well as to the participant’s state of mind or conditions (some subjects more than others can experience fatigue, loss of concentration, and oscillations in the task performance) [10,38,39]. Multivariate connectivity estimators, when applied to a multi-trial dataset, do not provide a connectivity pattern for each realization but an average pattern including all the aspects in common to all trials. In fact, it is well known that the estimation of a high number of parameters, as in MVAR-based approaches, require a high amount of data, a condition not satisfied at single-trial level. The lack of testing datasets reproducing inter-trial variability of the underlying pattern prevented the evaluation of the effects of this phenomenon in the accuracy of MVAR-based estimators.

Similar studies have already been proposed on small models (less than 10 nodes) [10], but only by employing this new toolbox were we able to simulate datasets reproducing standard EEG montages with higher numbers of electrodes in a matter of seconds (e.g., 32, 60 signals). The proposed simulation studies demonstrated that the combination of PDC and asymptotic statistic for its validation is robust and reliable even in the presence of some non-idealities which are typical of the real datasets, especially when recorded during specific motor tasks or from patients’ populations. At the same time, the two studies proposed here contributed to define more specific boundaries of acceptable quality of the data, in terms of consistency across the trials, for the application of those methods. If more than 50% of the trials is artifactual or shows a low signal to noise ratio, it is important to consider a significant loss of accuracy, as the actual brain network might be hidden by spurious links. The contribution of this application is twofold: on one hand, it returned a measure of the robustness of PDC to non-ideality in the data that can be easily encountered in many applications, and on the other, it showed how SEED-G toolbox can allow and support the evaluation of connectivity estimators under challenging experimental conditions, not yet rigorously tested.

## 6. Conclusions

SEED-G toolbox was developed with the aim to provide a useful instrument addressed to researchers in the field of brain connectivity. It allows for testing stationary and time-varying estimators in controlled and customizable, non-ideal conditions: low number of available samples and trials, high inter-trial variability, low signal to noise ratio. The application proposed in this study uncovered information about the optimal terms of use of the PDC and the errors to consider before making inferences for different levels of inconsistency across trials. Numerous other experimental conditions and connectivity methods could be studied using this open-source toolbox. Future works could investigate factors that play an important role in the accuracy of the connectivity estimates (e.g., optimal model order, algorithm for VAR model estimation, preprocessing procedures, linear inverse estimation), or even compare different algorithms under specific non-ideal experimental conditions. Therefore, we hope and believe that it will be helpful for researchers who want to test and develop new connectivity algorithms, and for making advancement in this fascinating research field.

## Figures and Tables

**Table 1:** Results of the four-way ANOVA (F-value) performed on the Relative Error evaluated after estimating PDC values on 5-, 10- and 20-node models.

	5 Nodes	10 Nodes	20 Nodes
VAR_DIR	98	162	15
VAR	995	2992	7275
MOD_CON	1237	4290	10176
TRIALS	1415	2860	6190
VAR × VAR_DIR	5	33	904
MOD_CON × VAR_DIR	2.91^(NS)^	22	558
MOD_CON × VAR	340	1806	5205
TRIALS × VAR_DIR	37	87	45
TRIALS × VAR	756	1676	4061
TRIALS × MOD_CON	690	1994	6458
MOD_CON × VAR × VAR_DIR	13	167	1361
TRIALS × VAR × VAR_DIR	8	28	539
TRIALS × MOD_CON × VAR_DIR	4	24	376
MOD_CON × VAR × TRIALS	132	405	1345
MOD_CON × VAR × TRIALS × VAR_DIR	4	39	322

^NS^ Factor of the ANOVA non-significant. When nothing is specified, *p* < 0.001.

**Table 2:** Results of the three-way ANOVA (F-value) performed on the performance parameters FPR and FNR, evaluated after estimating and validating PDC matrices.

	FPR *	FNR *
MOD_SIZE	740.7	251.1
SPURIOUS	237.3	118.7
TRIALS	77.3	189.4
SPURIOUS × MOD_SIZE	186.8	88.0
TRIALS × MOD_SIZE	39.6	149.3
SPURIOUS × TRIALS	4.2	50.7
SPURIOUS × TRIALS × MOD_SIZE	6.8	33.6

* All the factors of the ANOVA and their interactions are significant (*p* < 0.001).

## Data Availability

The data presented in this study are available on request from the corresponding author.

## Supplementary Materials

The supplementary materials are available online at https://www.mdpi.com/article/10.3390/s21113632/s1, including Figure S1: SEED-G performances in terms of extra required iterations, Figure S2: SEED-G extra required iterations and computational time when 20% of AR real components is included in the model, Figure S3: SEED-G extra required iterations and computational time when 30% of AR real components is included in the model.

## Appendix A

SEED-G toolbox was developed in MATLAB environment (tested on version R2017a and R2020b) and released on the GitHub page https://github.com/aanzolin/SEED-G-toolbox (accessed date 12 April 2021). It is organized in the following subfolders:

- **main**: it is the core of the toolbox and contains all the functions for the generation of EEG data according to a predefined ground-truth network.
- **dependencies**: containing parts of other toolboxes required to successfully run SEED-G functions. The links to the full packages can be found in the documentation on the GitHub page. The additional packages are Brain Connectivity Toolbox (BCT) [40], FieldTrip [41], Multivariate Granger Causality Toolbox (MVGC) [24], and AsympPDC Package (PDC_AsympSt) [42,43]. Additionally, the implemented forward model is solved according to the New York Head (NYH) model, whose parameters are contained in the structure available on the ICBM-NY platform [28].
- **real data**: containing real EEG data acquired from one healthy subject during resting state at scalp level (‘EEG_real_sources.mat’) and its reconstructed version in source domain (‘sLOR_cortical_sources.mat’). These signals can be employed to extract the AR components to be included in the model to generate data with the same spectral properties of the real ones.
- **demo**: containing examples of MATLAB scripts to be used to learn the different functionalities of the toolbox. For example, the code ‘run_generation.m’ allows to specify the directory containing the real sources and each specific input of the function ‘simulatedData_generation.m’.
- **auxiliary functions**: containing either original MATLAB functions or modified version of free available functions.
