# Supplementary material for: SEED-G: Simulated EEG Data Generator for Testing Connectivity Algorithms
Source: Sensors (Basel). 2021 May 23;21(11):3632. doi: 10.3390/s21113632 (PMC8197139; doi:10.3390/s21113632)
Supplement: Supplementary file 1 [file sensors-21-03632-s001.zip › sensors-1189158-supplementary.pdf]

sensors

Supplementary Material

SEED-G toolbox performances

The performance parameter Extra Required Iterations (ERIt) is defined as the number of rejected simulated datasets before completing the generation, normalized by the maximum number of allowed iterations that we imposed equal to 1000. ERIt is equal to 0% if the dataset is generated without extra iterations and it is equal to 100% when the dataset was generated using the maximum number of iterations. Figure S1 shows this measure as a function of Network Density and Model Size after imposing the number of AR components (Real Sources) included in the model equal to 30% of the generated signals. In the majority of cases, no extra iterations are required. For the generation of 32 time series with 30%-dense connectivity patterns, we found that 10% of extra iterations is on average required. The most challenging case to simulate was the one including 60 pseudo-EEG signals connected by a statistical network whose density was greater or equal than 20%. Such dataset was not generated even after using all the 1000 attempts.

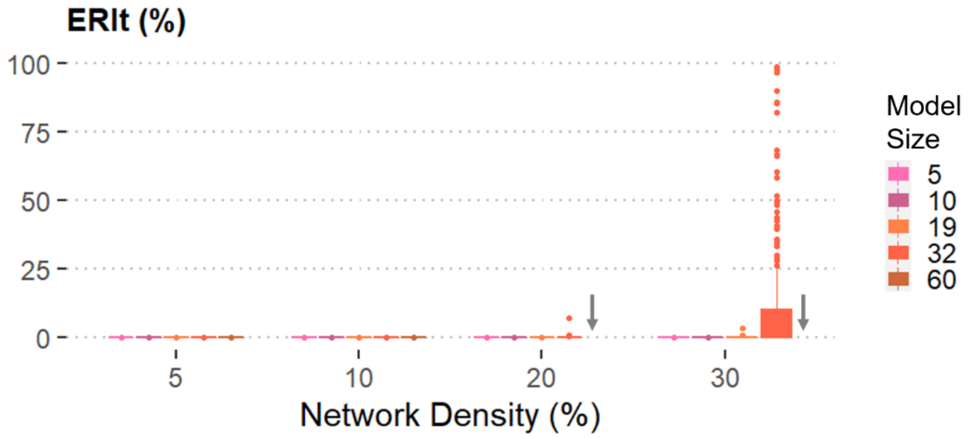

Figure S1. SEED-G performances in terms extra required iterations. Mean and standard deviation of the parameter ERIt computed over 300 iterations were showed as functions of the number of time series (Model Size) and the number of existing connections (Network Density). The number of real sources included is equal to 30%. The arrows indicate simulated conditions where the dataset was not generated.

Similar results were obtained changing the percentage of real sources in the model to 20% and 50% of the total number of time series included in the generated dataset, demonstrating that the number of real sources did not influence the number of extra iterations and the computational time required by the proposed toolbox for the generation process. In particular: Figure S2 reported the performance parameters for Real Sources equal to 20% and Figure 3S reported the performance parameters for Real Sources equal to 50%.

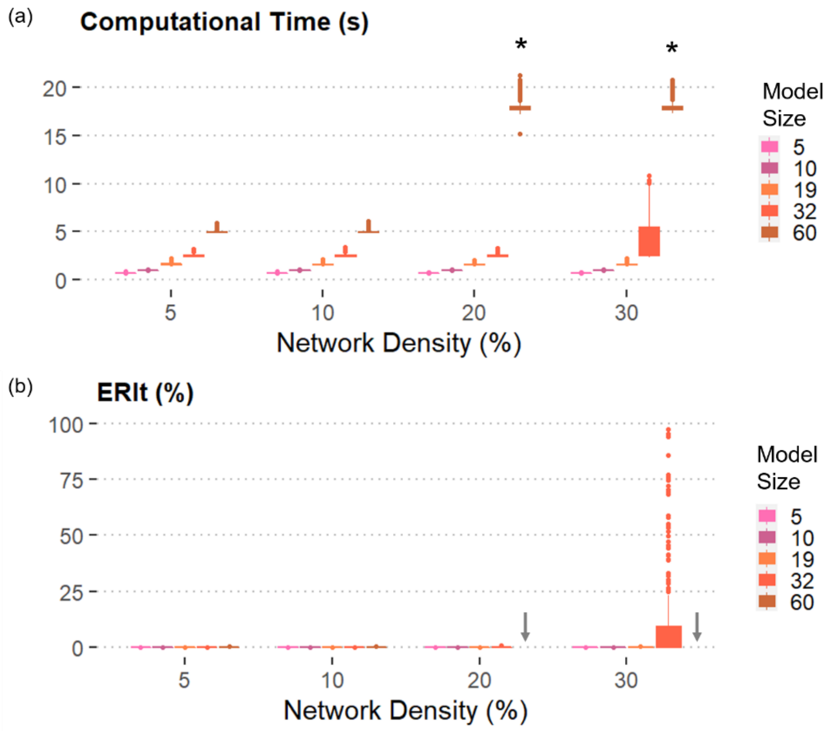

Figure S2. SEED-G extra required iterations and computational time when 20% of AR real components is included in the model. Mean and standard deviation of the parameter Computational Time (panel a) and ERIt (panel b) computed over 300 iterations were showed as functions of the number of time series (Model Size) and the number of existing connections (Network Density). The number of real sources included is equal to 20%. The asterisk as well as the arrows encode for the fact that no dataset with that specific combination of features could be generated with 1000 attempts.

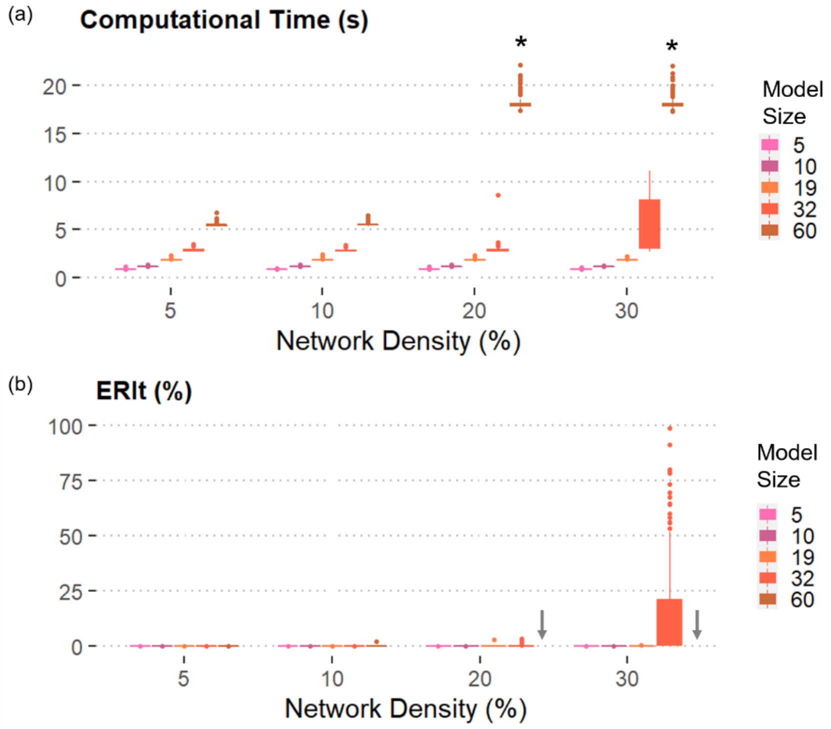

Figure S3. SEED-G extra required iterations and computational time when 30% of AR real components is included in the model. Mean and standard deviation of the parameter Computational Time (panel a) and ERIt (panel b) computed over 300 iterations were showed as functions of the number of time series (Model Size) and the number of existing connections (Network Density). The number of real sources included is equal to 30%. The asterisk as well as the arrows encode for the fact that no dataset with that specific combination of features could be generated with 1000 attempts.
